# Isolation-free detection of extracellular vesicles using Janus particles

**DOI:** 10.20517/evcna.2026.71

**Published:** 2026-07-09

**Authors:** Rienk Nieuwland, Edwin van der Pol, Agustin Enciso-Martinez

**Affiliations:** ^1^Department of Laboratory Medicine, Laboratory of Experimental Clinical Chemistry, Laboratory Specialized Diagnostics & Research, Amsterdam UMC, University of Amsterdam, Amsterdam 1105 AZ, The Netherlands.; ^2^Department of Biomedical Engineering & Physics, Amsterdam Cardiovascular Sciences, Cancer Center Amsterdam, Amsterdam UMC, University of Amsterdam, Amsterdam 1105 AZ, The Netherlands.; ^3^Oncode Institute and Department of Cell and Chemical Biology, Leiden University Medical Center, Leiden 2333 ZA, The Netherlands.

## MAIN TEXT

Clinical decision-making increasingly relies on biomarkers measured in body fluids that enable minimally invasive assessment of disease. Extracellular vesicles (EVs) are promising biomarkers in so-called liquid biopsies because they carry molecular cargo from their cells of origin. Unfortunately, disease-relevant EV populations are only a small fraction of the total EV pool, making their robust and reproducible analysis challenging. Detection of disease-relevant EVs directly in liquid biopsy samples, such as blood plasma or urine, is further complicated by the presence of non-vesicular extracellular particles (NVEPs) and soluble proteins, which often require time-consuming separation or enrichment steps. A recent study by Kumar and colleagues^[1]^ addresses this analytical problem by rethinking the detection step itself.

Kumar and colleagues introduce micrometer-sized polystyrene Janus particles, which are spheres with two distinct hemispheres [Figure 1]. One hemisphere is coated with gold and functionalized with antibodies to capture EVs of interest, whereas the other serves as an optical window that allows fluorescent light to escape the particles and generate the readout^[1]^. Due to Brownian rotation, the Janus particles alternately present their fluorescent and gold-coated hemispheres to the detector, causing them to blink under a fluorescence microscope. The blinking period increases with the size of the captured particle and can be measured with a low-magnification fluorescence microscope. Combining immunocapture of EVs with an optical readout of the Brownian rotation is the central conceptual advance of the study. Earlier studies showed that Janus particles can be useful for capturing and sensing EVs in body fluids^[2,3]^, and the work of Kumar and coworkers further paves the road towards their practical use^[1,4]^.

**Figure 1:**
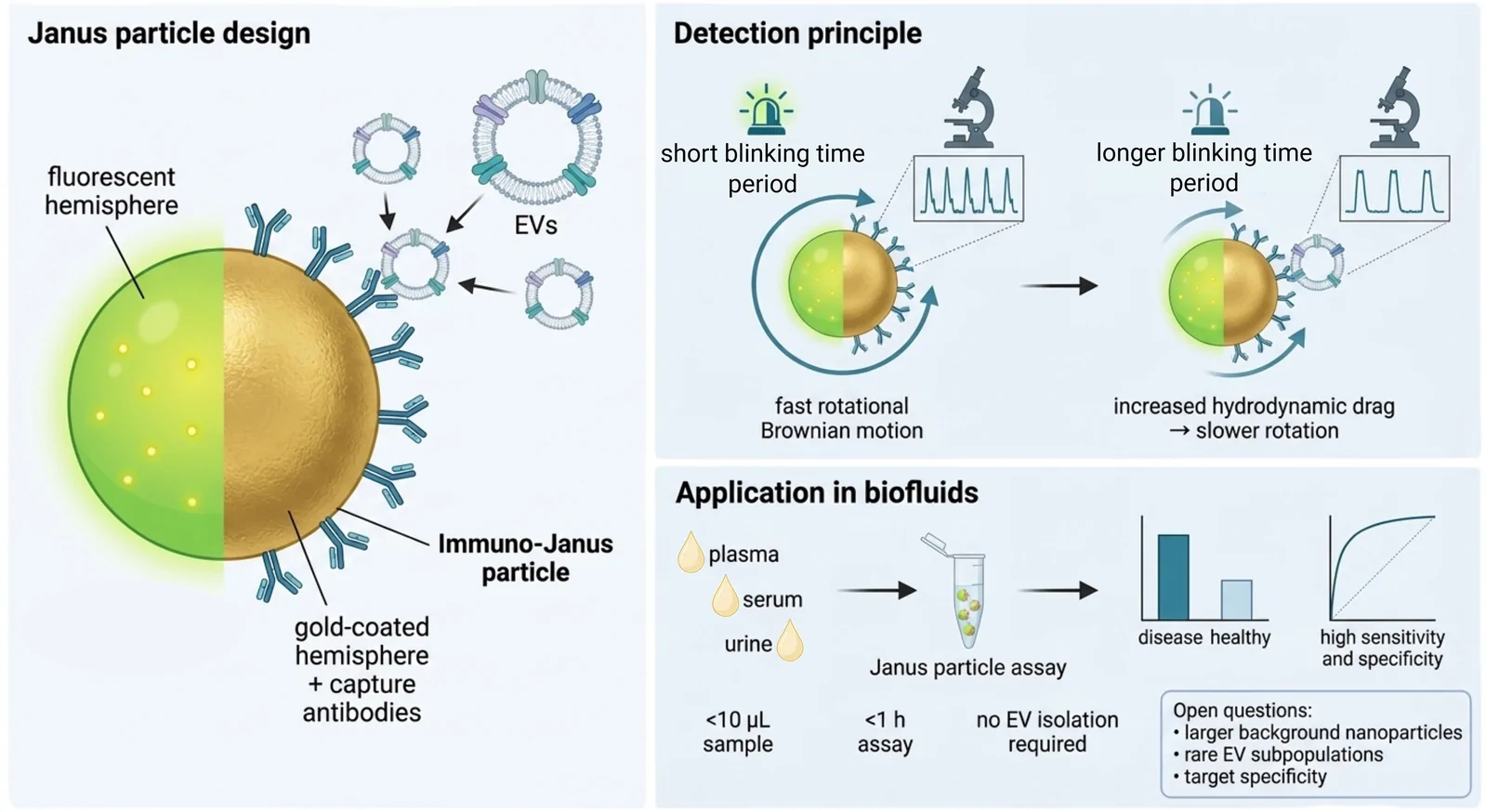
Combined capture of EVs and detection using immuno-Janus particles. Created in BioRender. Nieuwland, R. (2026) https://BioRender.com/qlv71u6. EVs: Extracellular vesicles.

Why do Janus particles matter for EV research? Firstly, the method combines what are usually separate steps. Instead of first isolating or enriching EVs and then measuring them, the Janus particles both capture target EVs and report their binding through altered blinking. The assay can be used in small sample volumes and is completed within one hour.

A second feature of the method is that binding of particles with a diameter of about 50 nm or less does not measurably perturb the blinking signal^[1]^. Soluble proteins can bind to the particle surface, but they are too small to meaningfully alter the blinking period. These points matter because the abundant presence of small NVEPs and soluble proteins is a major reason why EV workflows traditionally rely on pre-enrichment. By making the signal dependent on bound particles large enough to change the rotational drag, the Janus particle assay is less affected by small soluble components that otherwise can interfere with conventional assays. Collectively, this approach enables fast and isolation-free detection by combining capture and readout within a single platform, while reducing interference from soluble components.

The study is also notable for testing multiple clinically relevant biofluids and diseases. Kumar and colleagues validate the platform in multiple ways, including orthogonal comparison with ultracentrifugation plus surface plasmon resonance. They report direct use across plasma, serum, urine, and conditioned cell media, and apply the assay in a blind study of 87 samples from patients suffering from Alzheimer’s disease, colorectal cancer, glioblastoma, pancreatic ductal adenocarcinoma, and healthy controls. In that setting, disease groups were separated with sensitivity and specificity up to 0.99, depending on marker and comparison.

At the same time, the study raises an important question about rare-event detection. The Janus particle assay measures ensemble-average blinking across many particles rather than measuring single EVs. This improves the robustness of the assay, but also means that the assay reports population-level shifts rather than rare single binding events. This matters because the most clinically informative EVs may be scarce, with concentrations < 10 µL^-1^, particularly in early disease^[5]^. The assay clearly achieves a strong analytical sensitivity at the bulk level, but an important next question is how well that sensitivity translates to genuinely rare disease-associated EV populations when they occur within a much larger background of non-disease EVs.

A second question concerns the assay’s effective size window. Particles below ~50 nm appear not to contribute measurably to the Janus blinking signal, and the controls argue against a strong nonspecific blinking background in the tested samples. However, plasma also contains larger non-EV particles that are within the assay’s detectable size range. The study does not directly resolve the extent to which such particles may contribute to the readout, especially when they outnumber disease-relevant EV populations.

A related but distinct issue is specificity. The method is best understood as a powerful detection technology rather than a complete solution to disease-specific EV identification. Improved detection alone does not resolve the challenge of defining which EV populations are biologically or clinically relevant. Biofluids contain heterogeneous mixtures of EVs and NVEPs, and many commonly used markers are not uniquely disease-specific but overexpressed relative to normal cells and tissues. The proof-of-concept applications in this study use biologically relevant markers, including conformationally active epithelial growth factor receptor, carcinoembryonic antigen (CEA), glypican-1 (GPC1) and phosphorylated Tau181 (pTau181)^[1]^, but marker-positive signals do not automatically imply disease specificity. As the authors note, the method remains most informative when the chosen target is strongly associated with the EV population of interest, and identifying tumor-specific targets and capture reagents remains an important task. More broadly, a limitation of all antibody-based assays is their dependency on the specificity and consistency of the antibody used, such as clone selection and lot-to-lot variation. Thus, the Janus approach represents an important advance in detection, but the biological meaning and clinical value of the signal will depend on which particles are captured.

Ultimately, Kumar and colleagues show that EV detection can be made faster, less isolation-dependent, and compatible with minimal sample processing by exploiting a binding-induced change in measurable particle dynamics. The platform is innovative, validated within the scope of the paper, and directly relevant to one of the most persistent technical bottlenecks in the EV field. The next phase will be to define its operating boundaries more precisely, i.e., its sensitivity to rare and disease-specific EV subpopulations, its performance in the presence of larger NVEPs, and its compatibility with more selective molecular targets across broader clinical cohorts. Clinical translation will also depend on scalable and reproducible production of Janus particles, as well as robust and transferable data-analysis workflows. If these challenges can be addressed, Janus particles could offer a practical route toward faster and more scalable EV analysis in liquid biopsies.

## DECLARATIONS

### AI and AI-assisted tools statement

During the preparation of this manuscript, the AI tool ChatGPT (version 5.4, released 2026-03-05) was used solely for language editing. The tool did not influence the study design, data collection, analysis, interpretation, or the scientific content of the work. All authors take full responsibility for the accuracy, integrity, and final content of the manuscript.

### Copyright

© The Author(s) 2026.
